# Case Report: Arthroscopic management of a giant Baker cyst containing numerous rice body-like loose bodies in a patient with rheumatoid arthritis

**DOI:** 10.3389/fsurg.2026.1934749

**Published:** 2026-09-17

**Authors:** Yang Li, Yong Cao, Hangyu Li, Jinxin Gu, Jiasheng Ma, Linlin He, Xiaojun Ma, Jun Wan

**Affiliations:** 1 The Third Clinical Medical College, Ningxia Medical University, Yinchuan, China; 2 Department of Sports Medicine, People's Hospital of Ningxia Hui Autonomous Region, Ningxia Medical University, Yinchuan, Ningxia Hui Autonomous Region, China

**Keywords:** arthroscopy, Baker cyst, chronic synovitis, rheumatoid arthritis, rice body-like loose bodies

## Abstract

**Background:**

Giant Baker cysts containing numerous rice body-like loose bodies are uncommon and can resemble vascular, infectious, or neoplastic lesions.

**Case presentation:**

A 30-year-old woman with a 4-year history of rheumatoid arthritis presented with 1 year of progressive left knee pain, restricted flexion, and a 15 × 8 cm nonpulsatile mass extending from the popliteal fossa into the proximal calf. Magnetic resonance imaging showed a well-defined cystic lesion containing numerous nonmineralized intraluminal nodules. Arthroscopy demonstrated diffuse hypertrophic synovitis and multiple smooth ivory-white loose bodies. Synovectomy, loose-body removal, cyst decompression, and debridement of the cyst-joint communication were performed. Histology of the cyst wall and synovium showed chronic inflammation and fibrosis, without malignancy, cartilaginous metaplasia, granulomatous inflammation, or caseous necrosis. No isolated loose body had been separately embedded or sectioned. Results of interferon-gamma release assay, Ziehl–Neelsen staining, mycobacterial culture, and molecular testing were unavailable for retrospective review. At 12 months, the swelling had resolved, flexion improved from 110°to 130°, and ultrasonography showed no clinically significant recurrence of the popliteal cyst.

**Conclusion:**

Arthroscopy may provide tissue diagnosis and allow treatment of associated synovitis and the cyst–joint communication in selected patients. Detailed documentation of operative access and microbiological assessment is important for interpretation and reproducibility.

## 1 Introduction

A Baker cyst is a distension of the gastrocnemius-semimembranosus bursa that commonly communicates with the knee joint. In adults, it is usually associated with intra-articular disease, including osteoarthritis, meniscal pathology, and inflammatory arthritis. Persistent synovitis and joint effusion can drive fluid through the communication and progressively enlarge the bursa (1, 2).

Rheumatoid arthritis (RA) is a recognized setting for Baker cyst formation and for rice body formation. Rice bodies are small, smooth, free-floating structures composed mainly of fibrinous and collagenous material; they occur most often in chronic inflammatory synovitis but may also accompany tuberculous or other indolent infections (3–6). When numerous bodies are present in a large popliteal cyst, magnetic resonance imaging (MRI) may resemble synovial chondromatosis, tenosynovial giant cell tumor, chronic infection, hematoma, or soft-tissue neoplasia (5–8).

We describe a giant Baker cyst extending from the popliteal fossa into the proximal calf in a young woman with RA. The report focuses on the correlation among clinical, imaging, arthroscopic, and pathological findings, while explicitly addressing the limits of the available microbiological, histological, and operative documentation.

## 2 Case presentation

### 2.1 Clinical presentation and laboratory assessment

A 30-year-old woman with a 4-year history of RA presented with progressive left knee pain, posterior knee and proximal calf swelling, and activity-related limitation for approximately 1 year. She had received oral antirheumatic medication before admission, although the exact regimen was unavailable for retrospective verification. There was no documented acute trauma or previous knee surgery.

Examination showed mild knee swelling and a large, nonpulsatile cystic mass in the popliteal fossa and proximal calf, measuring approximately 15 × 8 cm. The skin was intact without erythema, ulceration, or sinus formation. Knee motion was 0–110°. Distal perfusion, sensation, skin temperature, and active ankle motion were preserved (Figure 1).

**Figure 1:**
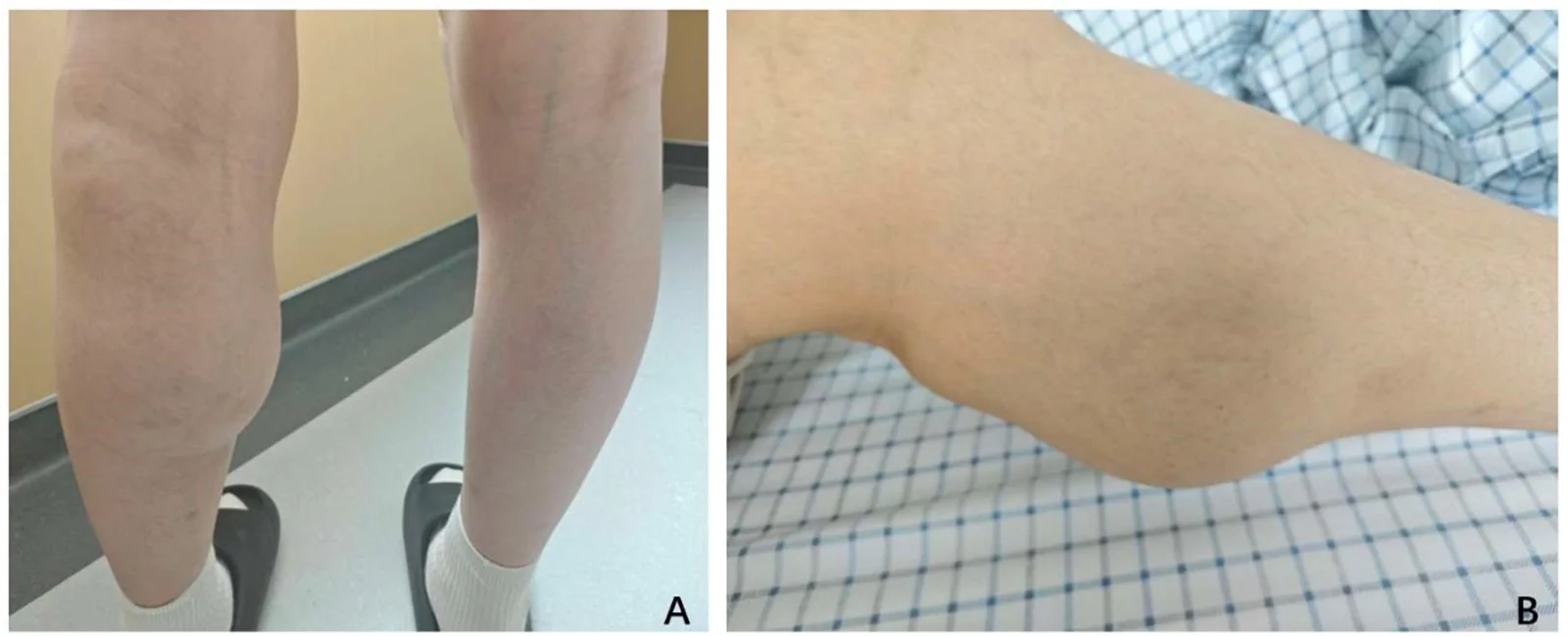
Preoperative clinical photographs. **(A)** Posterior view showing marked swelling of the left popliteal fossa and proximal calf. **(B)** Lateral view showing fusiform tumor-like swelling over the posterior knee; the skin was intact.

The erythrocyte sedimentation rate, C-reactive protein, rheumatoid factor, and anti-cyclic citrullinated peptide antibody level were elevated; the white blood cell and neutrophil counts were normal (Table 1). Although the D-dimer level was elevated, duplex ultrasonography showed patent and compressible deep veins without evidence of deep venous thrombosis. Cross-sectional imaging demonstrated a large cystic popliteal lesion. No results of interferon-gamma release assay, Ziehl-Neelsen staining, mycobacterial culture, mycobacterial polymerase chain reaction, or comprehensive bacterial and fungal cultures were available in the record reviewed for this report. Accordingly, chronic infection, including tuberculosis, could not be excluded microbiologically.

**Table 1:** Preoperative laboratory tests.

Test item	Result	Reference range	Interpretation
White blood cell count	6.12 × 10^9^/L	3.5–9.5 × 10^9^/L	Normal
Neutrophil count	3.85 × 10^9^/L	1.8–6.3 × 10^9^/L	Normal
Hemoglobin	102 g/L	115–150 g/L	Decreased
Platelet count	357 × 10^9^/L	125–350 × 10^9^/L	Slightly increased
Erythrocyte sedimentation rate	47.25 mm/h	0–26 mm/h	Increased
C-reactive protein	21.2 mg/L	0–6 mg/L	Increased
Rheumatoid factor	99.90 IU/mL	0–20 IU/mL	Increased
Anti-cyclic citrullinated peptide antibody	64.40 U/mL	0–17 U/mL	Increased
Anti-keratin antibody	Negative	Negative	Negative
Antistreptolysin O	55. IU/mL	0–408 IU/mL	Normal
D-dimer	2.3 ug/mL	0–1 ug/mL	Increased

ESR, erythrocyte sedimentation rate; CRP, C-reactive protein.

### 2.2 Imaging assessment

Radiographs showed a large posterior soft-tissue shadow without fracture, aggressive bone destruction, periosteal reaction, or calcified loose bodies (Figure 2). Computed tomography confirmed a well-defined posterior soft-tissue lesion without cortical destruction or mineralized bodies (Figure 3).

**Figure 2:**
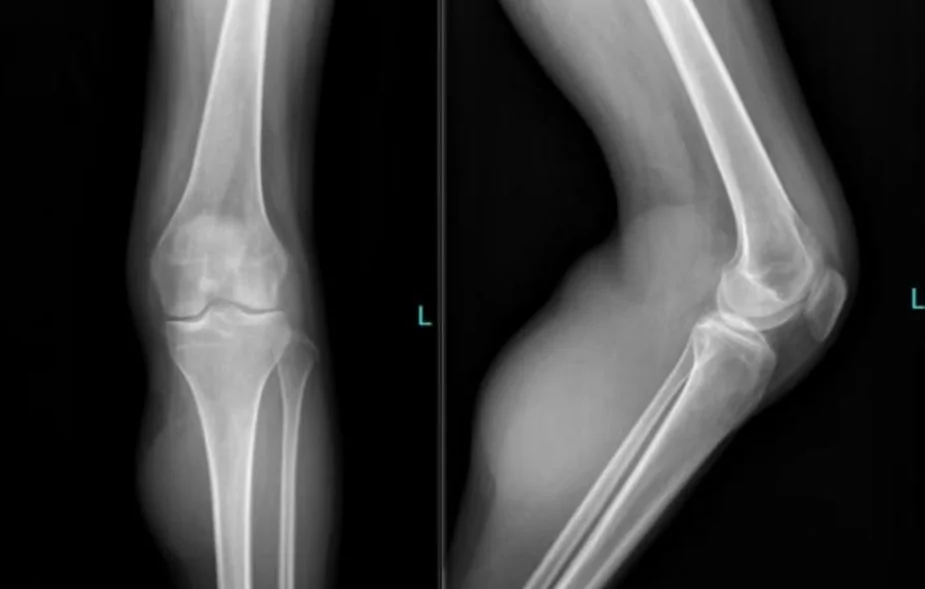
Preoperative anteroposterior and lateral radiographs of the left knee. A large posterior soft-tissue shadow is present without fracture, bone destruction, periosteal reaction, or calcified loose bodies.

**Figure 3:**
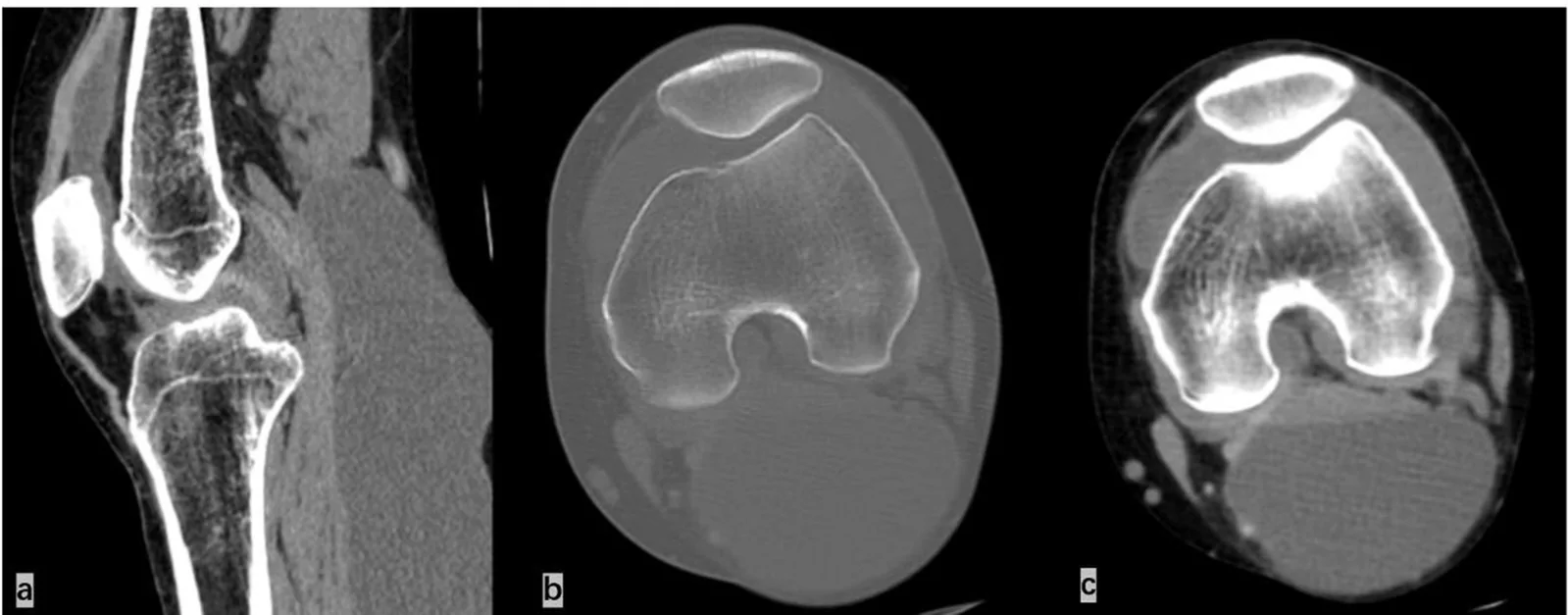
Computed tomography images of the left knee. **(a)** Sagittal image showing a large posterior knee soft-tissue lesion extending into the proximal calf. **(b,c)** Axial images showing no cortical destruction, periosteal reaction, or mineralized loose bodies.

MRI demonstrated a sharply marginated, fluid-filled lesion arising in the popliteal region and extending into the proximal calf. Numerous small low-signal intraluminal nodules were present, and the lesion displaced adjacent muscles without infiltrative growth or bone involvement. These features favored a giant Baker cyst with rice body-like loose bodies rather than a primary solid tumor (Figure 4).

**Figure 4:**
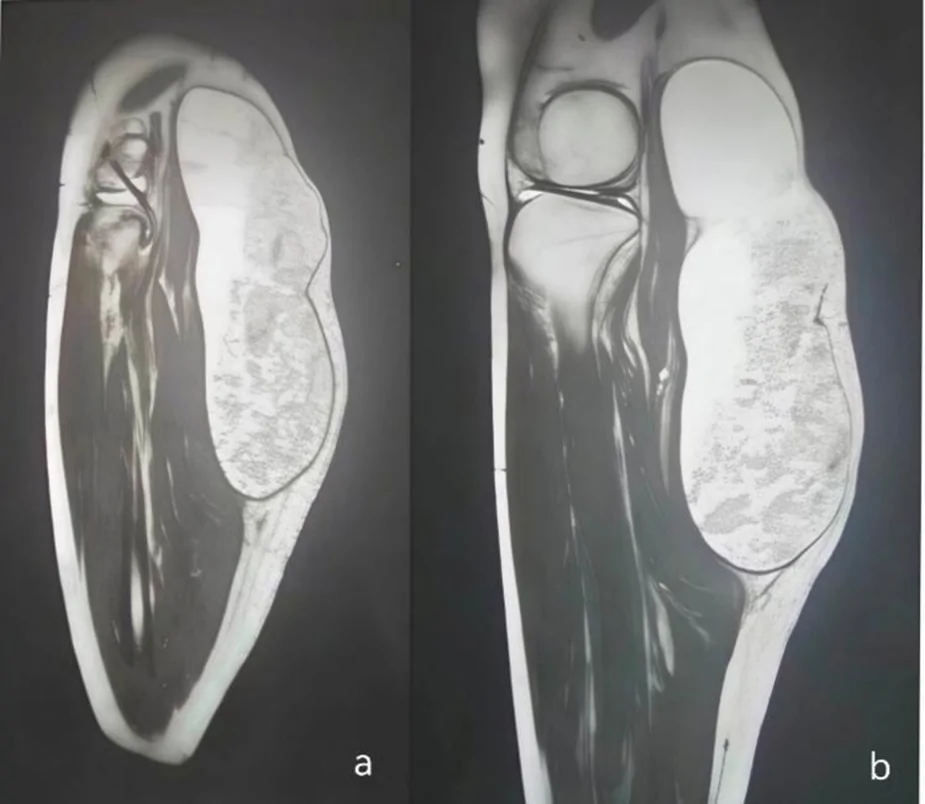
Magnetic resonance imaging of the giant popliteal cyst. **(a)** Axial image showing a well-defined fluid-filled lesion displacing adjacent musculature without infiltrative growth. **(b)** Sagittal image showing extension from the popliteal fossa into the proximal calf and numerous low-signal intraluminal nodules consistent with rice body-like loose bodies.

### 2.3 Arthroscopic treatment, pathology, and outcome

Surgery was undertaken because the lesion was large, symptomatic, and tumor-like, and because tissue diagnosis and treatment of the associated synovitis were required. Only standard anterolateral and anteromedial portals were documented in the available operative record. Arthroscopy showed hypertrophic and congested synovium in the suprapatellar pouch, intercondylar notch, and posterior compartment, together with numerous smooth, oval, ivory-white loose bodies (Figure 5). The operative note recorded debridement of hypertrophic synovium, enlargement of the cyst–joint communication, evacuation of cystic fluid and loose-body material, and extensive lavage. However, the exact route used to access the posteromedial compartment, the arthroscope angle, and the loose-body retrieval instruments could not be reconstructed retrospectively. Accordingly, the procedure is best described as arthroscopic decompression and debridement rather than complete cyst-wall excision.

**Figure 5:**
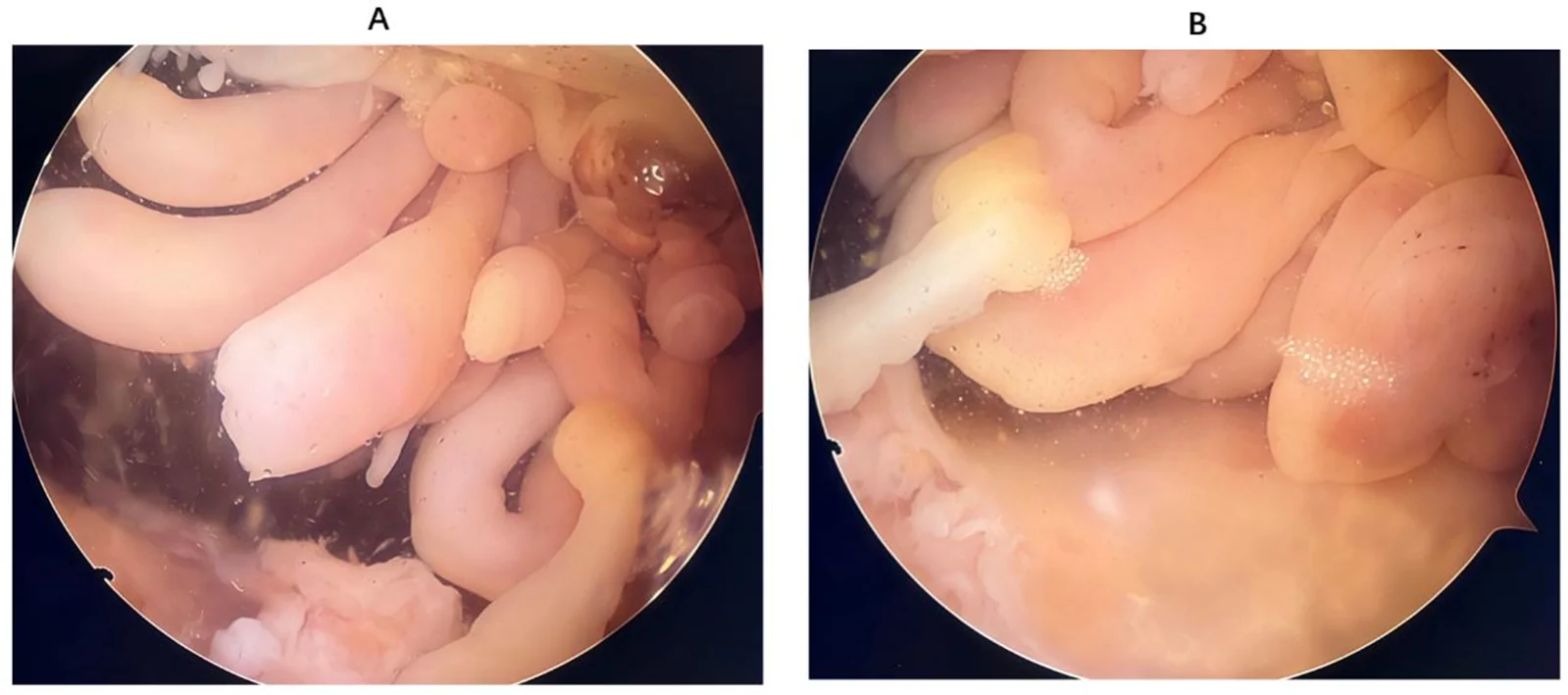
Intraoperative arthroscopic findings. **(A,B)** Numerous smooth, oval, ivory-white rice body-like loose bodies visualized during arthroscopy.

According to the operative record, cyst-wall tissue, synovial tissue, and loose-body material were submitted for pathological assessment. Available sections showed dense fibrous connective tissue with chronic inflammatory infiltration, vascular proliferation and congestion, and focal hyalinized degeneration (Figure 6). However, the archived slides available for retrospective review contained cyst-wall and synovial tissue only; no isolated loose body had been separately embedded or sectioned. Therefore, the bodies are described morphologically as rice body-like loose bodies rather than histologically confirmed rice bodies.

**Figure 6:**
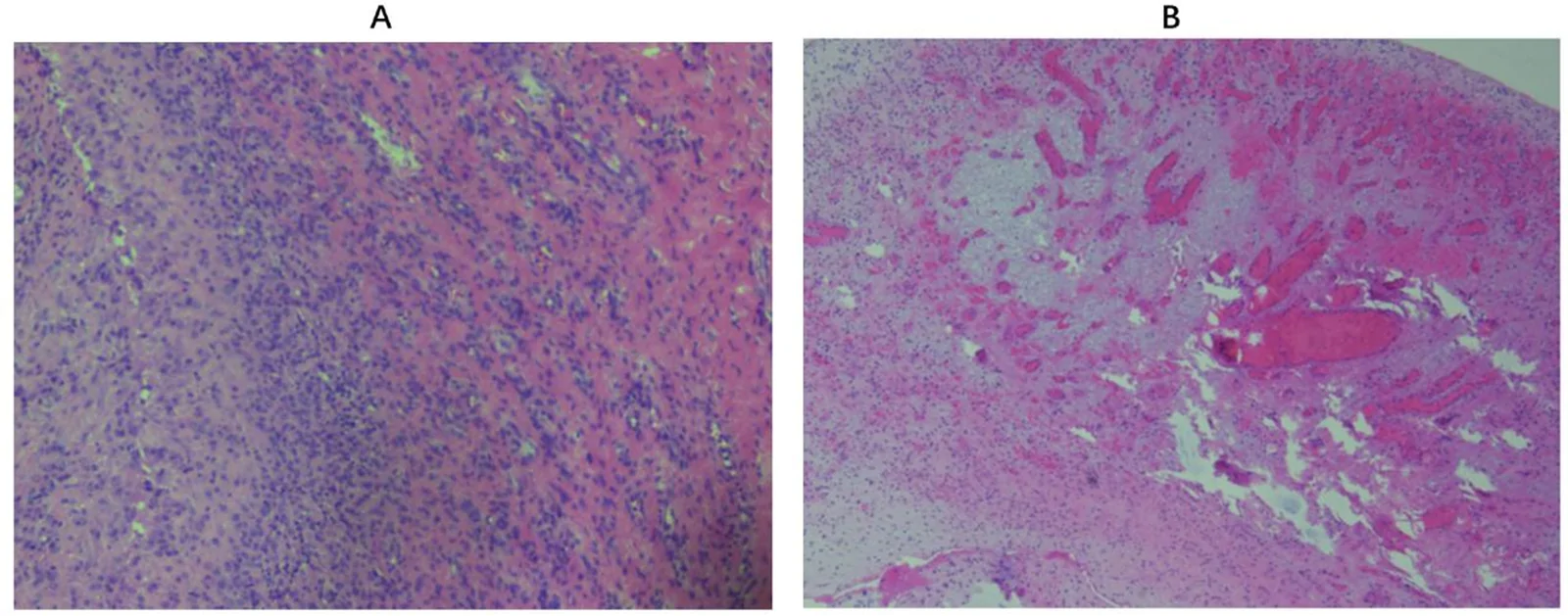
Histopathological examination. **(A)** Chronic inflammatory cell infiltration and fibrous synovial-like tissue proliferation. **(B)** Thickened fibrous cyst wall with hyalinized degeneration and vascular congestion; no malignancy, cartilaginous metaplasia, granulomatous inflammation, or caseous necrosis was identified. No isolated loose body was separately embedded or sectioned; therefore, its microscopic composition could not be directly confirmed.

Postoperatively, the patient received compression, limb elevation, analgesia, and progressive range-of-motion and quadriceps-strengthening exercises. Rheumatology follow-up was recommended to optimize disease-modifying antirheumatic treatment. At 12 months, pain and posterior swelling had substantially resolved, flexion improved from approximately 110°to 130° with full extension, and ultrasonography showed no clinically significant recurrence of the popliteal cyst. No wound, infectious, neurovascular, or thromboembolic complication was recorded.

### 2.4 Patient perspective

The patient reported that the operation relieved the posterior swelling and pain and allowed easier knee flexion during daily activities. She was satisfied with the functional improvement and agreed to continued rheumatology follow-up.

## 3 Discussion

In a patient with established RA and a chronic posterior knee or proximal calf mass, a complicated Baker cyst should be considered the leading diagnosis. Deep venous thrombosis, popliteal artery disease, chronic infection, and soft-tissue neoplasia remain important exclusions when the lesion is unusually large, rapidly progressive, painful, or otherwise atypical (Table 2) (7–11). In this case, the nonpulsatile examination, cystic MRI morphology, absence of destructive bone change, and inflammatory pathology supported a Baker cyst rather than a vascular or solid neoplastic lesion.

**Table 2:** Structured differential diagnosis.

Differential diagnosis	Reason for consideration	Findings in this case
Deep venous thrombosis	Unilateral calf swelling and elevated D-dimer	duplex ultrasonography showed patent and compressible deep veins without evidence of deep venous thrombosis.
Popliteal artery aneurysm	A posterior knee mass may be vascular and can compromise distal arterial flow	The mass was nonpulsatile, distal perfusion was preserved, and cross-sectional imaging demonstrated a cystic rather than vascular lesion.
Soft-tissue neoplasm	Large tumor-like mass extending into the proximal calf	MRI showed no solid infiltrative component or bone destruction, and pathology showed no malignancy.
Synovial chondromatosis	Numerous intracystic nodules may resemble loose bodies	Radiographs and CT showed no mineralization, and histology showed no cartilaginous metaplasia.
Diffuse-type tenosynovial giant cell tumor	Synovial proliferation may cause swelling and restricted motion	MRI and arthroscopy did not demonstrate typical hemosiderin-related low signal or brown villonodular synovial proliferation.
Tuberculous or other chronic infection	Rice bodies may occur in tuberculous or indolent infectious synovitis	No sinus, purulence, granulomas, or caseous necrosis was observed; however, dedicated microbiological results were unavailable.

The findings are biologically consistent with chronic rheumatoid synovitis. Ongoing inflammation increases synovial-fluid production and intra-articular pressure; fluid can then pass through the communication with the gastrocnemius-semimembranosus bursa, where a valve-like mechanism promotes progressive enlargement (1, 2, 12, 13). Fibrin aggregation, synovial debris, tissue degeneration, and repetitive motion may contribute to the formation of rice body-like material (3–5, 14). Because the cyst reflects active intra-articular disease, long-term control of RA is central to reducing recurrent effusion and cyst formation.

MRI was the most informative preoperative study because it defined the cystic nature, distal extension, internal nodules, and relationship to adjacent structures. Radiographs and computed tomography were useful for excluding calcified bodies and aggressive osseous change. Imaging, however, cannot determine the cause of rice body-like material. Tuberculous synovitis is a particularly relevant alternative because it may also produce rice bodies (6). The absence of granulomas and caseous necrosis reduced, but did not eliminate, this possibility. Since no interferon-gamma release assay, Ziehl–Neelsen staining, dedicated mycobacterial culture, or molecular testing result was available for retrospective review, tuberculosis could not be definitively excluded on microbiological grounds.

Arthroscopy can address intra-articular synovitis, remove loose bodies, decompress the cyst, obtain tissue, and treat the cyst-joint communication during the same procedure (15–19). Posteromedial or direct posterior portals may improve access in selected cysts, whereas open or combined surgery may be preferable for multiloculated lesions, major neurovascular compression, suspected neoplasia, or a need for complete cyst-wall excision (10, 15–19). In the present case, only standard anterior portals were documented. The lack of reliable information on any posterior portal, scope angle, and retrieval device limits technical reproducibility and prevents comparison of this portal strategy with other approaches.

The favorable 12-month clinical and ultrasonographic outcome suggests that decompression and treatment of the associated synovitis were clinically effective in this patient. It does not establish that arthroscopy is superior to open excision, nor does it exclude later recurrence. Continued rheumatologic management and longer surveillance remain appropriate.

## 4 Limitations

This report has several limitations. First, it describes a single patient and includes only 12 months of follow-up. Second, dedicated microbiological testing for tuberculosis and other indolent infections was unavailable; therefore, infection could not be definitively excluded microbiologically. Third, no isolated loose body was separately embedded or sectioned, preventing direct microscopic confirmation of its composition. In addition, the preoperative and postoperative disease-modifying antirheumatic regimen could not be fully verified, limiting assessment of the influence of systemic disease control on recurrence. Finally, the available operative record did not provide sufficient detail to verify the posterior access route, arthroscope angle, or loose-body retrieval method, which limits technical reproducibility.

## 5 Conclusion

A giant Baker cyst containing numerous rice body-like loose bodies can present as a tumor-like posterior knee and proximal calf mass in RA. In this clinical setting, a complicated Baker cyst should be considered early, while vascular, infectious, and neoplastic alternatives are actively excluded. MRI defines the lesion and its extent, and arthroscopy may permit tissue sampling, synovectomy, loose-body removal, cyst decompression, and treatment of the cyst-joint communication in selected patients. When rice body-like material is identified, microbiological investigation and direct histological assessment of the loose bodies should be pursued whenever feasible. Detailed documentation of the arthroscopic access route is also important for technical reproducibility.

## Data Availability

The original contributions presented in the study are included in the article/Supplementary Material, further inquiries can be directed to the corresponding author.
